# A GM-JMNS-CPHD Filter for Different-Fields-of-View Stochastic Outlier Selection for Nonlinear Motion Tracking

**DOI:** 10.3390/s24103176

**Published:** 2024-05-16

**Authors:** Liu Wang, Jian Zhao, Lijuan Shi, Yuan Liu, Jing Zhang

**Affiliations:** 1 The Key Laboratory of Intelligent Rehabilitation and Barrier-Free for the Disabled, Changchun University, Ministry of Education, Changchun 130012, China; wangl95@ccu.edu.cn (L.W.); shilj@ccu.edu.cn (L.S.); 220401152@mails.ccu.edu.cn (Y.L.); 220401143@mails.ccu.edu.cn (J.Z.); 2 Jilin Provincial Key Laboratory of Human Health Status Identification & Function Enhancement, Changchun 130000, China; 3 College of Computer Science and Technology, Changchun University, Changchun 130000, China; 4 College of Electronic and Information Engineering, Changchun University, Changchun 130000, China

**Keywords:** different fields of view, GM-JMNS-CPHD, stochastic outlier selection, nonlinear motion tracking

## Abstract

Most multi-target movements are nonlinear in the process of movement. The common multi-target tracking filtering methods directly act on the multi-target tracking system of nonlinear targets, and the fusion effect is worse under the influence of different perspectives. Aiming to determine the influence of different perspectives on the fusion accuracy of multi-sensor tracking in the process of target tracking, this paper studies the multi-target tracking fusion strategy of a nonlinear system with different perspectives. A GM-JMNS-CPHD fusion technique is introduced for random outlier selection in multi-target tracking, leveraging sensors with limited views. By employing boundary segmentation from distinct perspectives, the posterior intensity function undergoes decomposition into multiple sub-intensities through SOS clustering. The distribution of target numbers within the respective regions is then characterized by the multi-Bernoulli reconstruction cardinal distribution. Simulation outcomes demonstrate the robustness and efficacy of this approach. In comparison to other algorithms, this method exhibits enhanced robustness even amidst a decreased detection probability and heightened clutter rates.

## 1. Introduction

Distributed fusion offers several advantages, including minimal bandwidth demands for communication links, the ability of nodes to handle data from nearby nodes, decreased system computing power requirements, and the flexibility to relocate or remove sensor nodes for simplified management. Consequently, distributed sensor networks find extensive applications across diverse research domains like military defense, smart industry, and industrial automation. Engaging in relevant technological investigations holds significant scientific importance. Multi-target tracking, employing random finite set (RFS) technology, circumvents intricate data-association procedures and has emerged as a focal point in multi-target tracking research [1,2,3,4,5]. The theory of RFS provides a unified and comprehensive theoretical framework for multi-target tracking problems in complex and variable monitoring environments and is widely applied in the distributed fusion of multiple sensors [6,7,8,9,10,11,12,13,14,15]. The Cardinalized Probability Hypothesis Density (CPHD) filter in random finite set theory is widely used due to its low computational cost and ability to avoid inconsistent label spaces [16,17,18,19,20].

In practical application scenarios, various sensor nodes in sensor networks have inconsistent and limited fields of view due to their different positions and angles, and different fields of view often correspond to different target state information. Directly using distributed fusion algorithms under different view conditions may not guarantee the good tracking performance of sensor networks. In other words, throughout the multi-sensor acquisition process, variations in perspectives among sensors may occur, including legitimate viewpoints and overlapping perspectives. There may be common information between these perspectives, which can lead to unknown correlations between multiple sensors and can affect fusion accuracy. At present, most research on distributed fusion algorithms assumes that the field of view between sensor nodes is consistent and can cover the entire monitoring area. It can be seen that the main research direction of current scholars is the impact of different perspectives on target tracking. Regarding the differences in perspective in sensors, Shen, et al. [21] introduced an innovative consensus-based labeled multi-Bernoulli (LMB) filter, which can effectively overcome the problem of label space mismatch in different sensor perspectives. Li, et al. [22,23] proposed a distributed fusion method for different sensor networks in different perspectives. The distributed fusion method proposed by Li, et al. [24] in his research is called local diffusion, which performs a neighbor communication iteration in either of two ways at each filtering step, realizing target tracking with perspective differences. Li, et al. [25] used labeled multi-Bernoulli (LMB) filters for multi-target tracking in different fields of view in his research. Yi, et al. [26] employed a series of dynamically calculated fusion weights in their research to conduct a weighted arithmetic average (WAA), enhancing robustness in the process, which is suitable for PHD filter-distributed multi-sensor fusion in different sensor fields of view. Da, et al. [27] proposed a novel GMP Jumping Markov CPHD (GMP-JMCPHD) filter implementation in his research to handle highly nonlinear/non-Gaussian models and target maneuvering. It is evident that the primary focus of current research is on optimizing fusion algorithms to address the challenges of multi-sensor and multi-target tracking from varying perspectives. However, the above methods are mainly applied to linear systems. In reality, multi-targets move nonlinearly during the motion process, and most of them move nonlinearly during the motion process, such as vehicle overspeed measurement and ship navigation. Nonlinear systems can more accurately describe the system’s motion state, while GM-PHD and GM-CPHD methods directly act on the multi-target tracking system of nonlinear targets, and the fusion effect is even worse under the influence of different perspectives.

Based on this, the focus of this study is to explore the multi-target tracking problem of distributed sensors in sensor networks with different FoVs. From the two aspects of moving-target state-space partitioning and fusion algorithms, the impact of different fields of view on nonlinear systems is verified. Suggesting a novel fusion strategy, the initial step involves decomposing the state space of fields of view (FoVs), followed by the utilization of the SOS clustering algorithm. This approach aims to enhance the performance of fusion criteria for tracking nonlinear motion targets observed from different perspectives.

The section arrangement of this article is as follows: Section 1, Introduction, introduces the research background of the article; Section 2, Research Background, introduces the impact of different fields of view on nonlinear systems and the GM-JMNS-CPHD filter and introduces the stochastic outlier selection algorithm; Section 3, Application of SOS-GM-JMNS-CPHD Algorithm to Nonlinear Systems, introduces the steps and processes of splitting, fusion, and merging; Section 4, Simulation Results, presents the application and analysis of SOS-GM-CPHD in nonlinear systems, a comparative analysis of SOS-GM-JMNS-CPHD and other algorithms, and algorithm complexity validation.

## 2. Research Background

### 2.1. Analysis of the Influence of Different Fields of View on Nonlinear Systems

**Figure 1:**
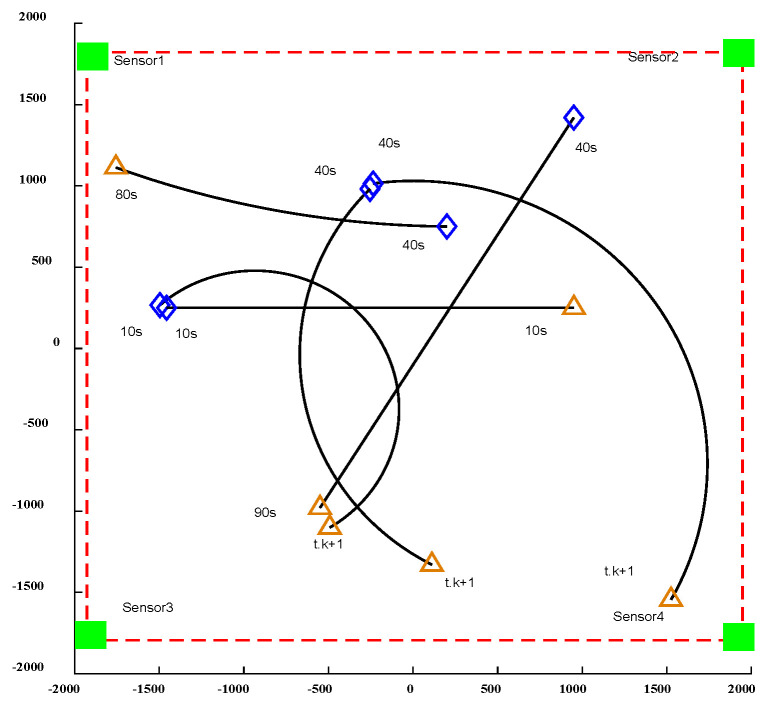
Generation time of moving object in nonlinear motion model.

Most multi-target movements are nonlinear in the process of movement. There is little existing research on non-uniform-field-of-view sensors, and there is even less research on multi-sensor tracking for non-uniform-field-of-view, nonlinear systems. In order to analyze the influence of non-uniform-field-of-view multi-sensors on nonlinear systems, the influence of different-diameter sensors in a nonlinear motion model on the multi-target tracking of nonlinear systems and the influence of different-angle sensors on the multi-target tracking of nonlinear systems is analyzed; as shown in Figure 1, the trajectory of a moving target in a nonlinear motion model and the birth time and death time of each trajectory are different.

**Figure 2:**
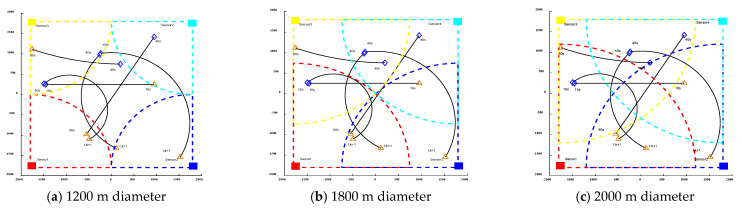
Influence of sensors with different detection diameters on multi-target tracking in nonlinear systems.

**Figure 3:**
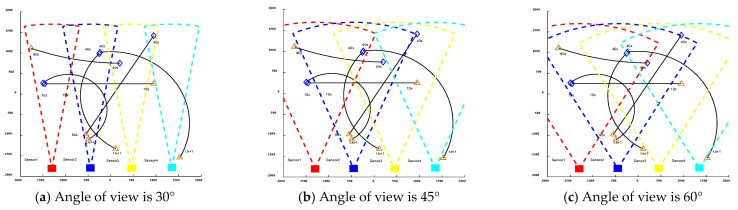
Influence of sensors with different detection angles on multi-target tracking in nonlinear systems.

Based on the motion trajectory of the above nonlinear motion model, sensors with different detection diameters and different detection angles are placed. Figure 2 and Figure 3 illustrate the influence of sensors with varying detection diameters and different detection perspectives on multi-target tracking in nonlinear systems. The effect of non-uniform fields of view on linear systems is comparable, as depicted in the figure. The trajectories of moving targets captured by sensors with different diameters and perspectives exhibit significant variations, thereby directly influencing the efficacy of moving-target acquisition.

**Figure 4:**
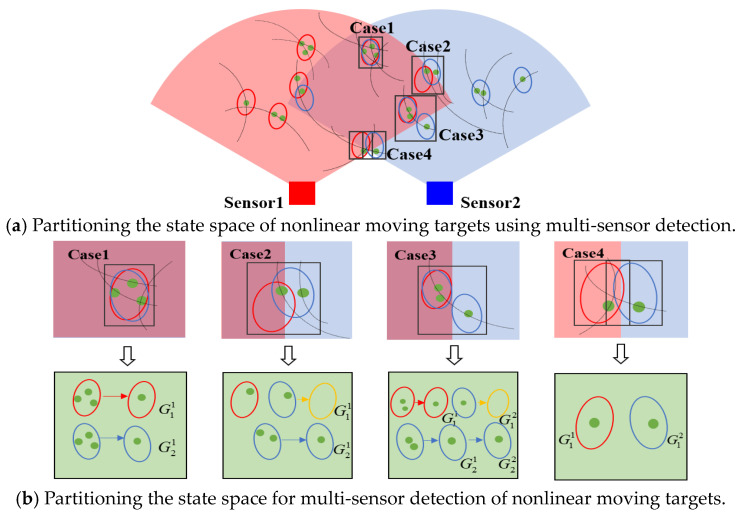
Results of partitioning the state space for multi-view multi-sensor detection of nonlinear moving targets.

As shown in Figure 4a, the FoV center angles are all 90°, and the sensor FoV is 60°. At this point, the sensors used to collect moving targets will be affected by their position and direction, resulting in differences in the acquisition perspective. Therefore, it is necessary to partition the state of nonlinear moving targets.

As shown in Figure 4b, the state-space division for nonlinear moving targets detected by multi-sensors also encounters challenges such as underestimating and overestimating the number of targets.

### 2.2. GM-JMNS-CPHD Filter

Regarding the CPHD (Cardinalized Probability Hypothesis Density) filter [28,29], the fusion method integrates the propagation intensity function and cardinality distribution, considering the clutter RFS (random finite set) as an Independent and Identically Distributed (IID) process. B.N. Vo, et al. [30] and R. Mahler, et al. [31,32] proposed the JM-CPHD filter in their study, which can be viewed as an extension of the state x integral in traditional CPHD filters to a double integral $\ddot{x}=(x,o)$ of mode and state.

When a JMNS (Jumping Markov Nonlinear System) is applied to CPHD filters, the model lacks a target-generation model, and both the target-generation and clutter models must incorporate the probability distributions $p_{\left(k+1\left|k\right.\right)}^{B}(n)$ and $p_{\left(k+1\right)}^{\kappa }(m)$ for the number of new targets and clutter measurements, respectively. These distributions must adhere to the following requirements [33,34,35,36,37]:

(1) $$\sum_{m\geq 0}n\cdot p_{(k+1)}^{B}(n)=\sum_{o}\int b_{\left(k+1\left|k\right.(x,o)\right)}dx$$

(2) $$\sum_{m\geq 0}m\cdot p_{\left(k+1\right)}^{\kappa }(n)=\lambda_{\left(k+1\right)}$$

For the probability distribution p(n) on a positive integer *n*, if n is negative, then p(n)=0. For the combination coefficient $C_{n,i}$, if i>n, then $C_{n,i}=0$. At this point, $\ddot{x}$ can be used instead of x.

(1) Prediction of JMNS-CPHD Filter

The prediction of the JMNS-CPHD filter can be expressed as follows:

(3) $$D_{\left(k+1\left|k\right.\right)}(\ddot{x})=b_{\left(k+1\left|k\right.\right)}(\ddot{x})+\int p_{s}({\ddot{x}}^{\prime })\cdot f_{\left(k+1\left|k\right.\right)}(\ddot{x}\left|{\ddot{x}}^{\prime }\right.)\cdot D_{\left(k+1\left|k\right.\right)}(\ddot{x})d\ddot{x}$$

Among them, $b_{\left(k+1\left|k\right.\right)}(\ddot{x})$ is the intensity function during the appearance of the target, $p_{s}({\ddot{x}}^{\prime })$ is the probability of target survival, and $f_{\left(k+1\left|k\right.\right)}(\ddot{x}\left|{\ddot{x}}^{\prime }\right.)$ is the Jumping Markov transition density.

(4) $$p_{\left(k+1\left|k\right.\right)}(n)=\sum_{n^{\prime }\geq 0}p_{\left(k+1\left|k\right.\right)}(n\left|n^{\prime }\right.)\cdot p_{\left(k\left|k\right.\right)}(n^{\prime })$$

The Markov transition probability $p_{\left(k+1\left|k\right.\right)}(n\left|n^{\prime }\right.)$ is as follows:

(5) $$p_{\left(k+1\left|k\right.\right)}(n\left|n^{\prime }\right.)=\sum_{l=j}^{\infty }C_{j}^{l}p(l)\frac{{\langle p_{\left(s,k\right)},D\rangle }^{j}{\langle 1-p_{\left(s,k\right)},D\rangle }^{j}}{{\langle 1,D\rangle }^{l}}$$

This can be rewritten using Jumping Markov notation as follows:

(6) $$D_{\left(k+1\left|k\right.\right)}(x,o)=b_{\left(k+1\left|k\right.\right)}(x,o)+\sum_{o^{\prime }}X_{\left(o,o^{\prime }\right)}\int p_{s}(x^{\prime },o^{\prime })\cdot f_{\left(k+1\left|k\right.\right)}(x^{\prime }\left|x^{\prime },o^{\prime }\right.)\cdot D_{\left(k+1\left|k\right.\right)}(x^{\prime },o^{\prime })dx^{\prime }$$

(2) Update of JMNS-CPHD filter

The update function of the PHD at time k, given the distribution of prediction cardinality $p_{\left(k+1\left|k\right.\right)}(n)$ and $D_{\left(k+1\left|k\right.\right)}(x,o)$, can be expressed as follows:

(7) $$p_{\left(k\left|k\right.\right)}(n)=\frac{b_{k}^{0}\left[D_{\left(k+1\left|k\right.\right)},Z_{k}\right](n)p_{\left(k+1\left|k\right.\right)}(n)}{\langle b_{k}^{0}\left[D_{\left(k+1\left|k\right.\right)},Z_{k}\right],p_{\left(k+1\left|k\right.\right)}\rangle }$$

(8) $$\begin{array}{l} D_{\left(k\left|k\right.\right)}(x,o)=(1-p_{\left(k\left|k\right.\right)}(x,o))\frac{\langle b_{k}^{1}\left[D_{\left(k+1\left|k\right.\right)},Z_{k}\right],p_{\left(k+1\left|k\right.\right)}\rangle }{\langle b_{k}^{0}\left[D_{\left(k+1\left|k\right.\right)},Z_{k}\right],p_{\left(k+1\left|k\right.\right)}\rangle }D_{\left(k+1\left|k\right.\right)}(x,o)\\ +\sum_{z\in Z_{k}}\frac{\langle b_{k}^{1}\left[D_{\left(k+1\left|k\right.\right)},\frac{Z_{k}}{\left\{z\right\}}\right],p_{\left(k+1\left|k\right.\right)}\rangle }{\langle b_{k}^{0}\left[D_{\left(k+1\left|k\right.\right)},Z_{k}\right],p_{\left(k+1\left|k\right.\right)}\rangle }\frac{\langle 1,\kappa_{k}\rangle g_{k}(z\left|x,o\right.)p_{\left(D,k\right)}(x,o)}{\kappa_{k}(z)}D_{\left(k+1\left|k\right.\right)}(x,o) \end{array}$$

Among them,

(9) $$\begin{array}{l} b_{k}^{u}\left[D,Z\right](n)=\sum_{j=0}^{\min(\left|Z\right|,n)}\left(\left|Z\right|-j\right)!p_{k}^{\kappa }(\left|Z\right|-j)P_{j+u}^{n}\\ \times \frac{{\langle 1-p_{D,k},D\rangle }^{n-(j+u)}}{{\langle 1,D\rangle }^{n}}e_{j}\left\{\langle D,\varphi_{k,z}\rangle :z\in Z\right\} \end{array}$$

$e_{j}(Z)$ represents an elementary symmetric function as follows:

(10) $$e_{j}(Z)=\sum_{S\subseteq Z,\left|S\right|=j}\prod_{i=S^{i}}i,e_{0}(Z)=1$$

(3) Prediction of GM-JMNS-CPHD Filter

Da in reference [27] proposed a new Gaussian Mixture Particle (GMP) and implemented Jumping Markov CPHD filter in his research to handle highly nonlinear/non-Gaussian models and target maneuvering. The principle is that particles achieve local filtering propagation and updates, while Gaussian distribution achieves communication and fusion. Assuming independence between target-survival probability and sensor-detection probability with respect to the state, they can be segregated into two distinct phases: prediction and update. The Gaussian distribution form of $b_{\left(k+1\left|k\right.\right)}(x,o)$ and $D_{\left(k-1\left|k\right.\right)}(x,o)$ for newly born goals is as follows:

(11) $$b_{\left(k+1\left|k\right.\right)}(x,o)=\sum_{j=1}^{J_{B,o}}b_{k}^{j}(o)N(x;m_{\left(B,k\right)}^{j}(o),P_{\left(B,k\right)}^{j}(o))$$

(12) $$D_{\left(k-1\left|k\right.\right)}(x,o)=\sum_{i=1}^{J_{\left(o,k-1\right)}}{D^{\prime }}_{\left(k-1\left|k\right.\right)}(o)N(x;m_{\left(k-1\right)}^{i}(o),P_{\left(k-1\right)}^{i}(o))$$

At this point, according to Formulas (31) and (32), it can be expressed as follows:

(13) $$p_{\left(k+1\left|k\right.\right)}(n)=\sum_{n^{\prime }\geq 0}p_{\left(k+1\left|k\right.\right)}(n\left|n^{\prime }\right.)\cdot {\bar{p}}_{\left(k\left|k\right.\right)}(n^{\prime })$$

The Markov transition probability ${\bar{p}}_{\left(k+1\left|k\right.\right)}(n\left|n^{\prime }\right.)$ is

(14) $${\bar{p}}_{\left(k+1\left|k\right.\right)}(n\left|n^{\prime }\right.)=\sum_{l=j}^{\infty }C_{j}^{l}p(l)\frac{\Gamma^{j}\langle p_{(s,k)},D\rangle \Gamma^{j}\langle 1-p_{(s,k)},D\rangle }{\Gamma^{l}\langle 1,D\rangle }$$

Among them,

(15) $$\Gamma \langle p_{\left(s,k\right)},D\rangle =\sum_{o}p_{s}(o)\sum_{i=1}^{J_{o,k-1}}{D^{\prime }}_{\left(k-1\left|k\right.\right)}^{i}(o)$$

This can be rewritten using Jumping Markov notation as follows:

(16) $$\begin{array}{l} D_{\left(k+1\left|k\right.\right)}(x,o)=b_{\left(k+1\left|k\right.\right)}(x,o)+\sum_{o^{\prime }}\sum_{i=1}^{J_{\left(o,k-1\right)}}X_{o,o^{\prime }}p_{s}(x^{\prime },o^{\prime })D_{\left(k-1\right)}^{i\prime }(x^{\prime },o^{\prime })\\ N(x;\frac{\sum_{j=1}^{M}x_{+}^{i,j}}{M},\frac{\sum_{j=1}^{M}\left[m_{\left(S,+\right)}^{i}(o^{\prime })-x_{+}^{i,j}\right]{\left[m_{\left(S,+\right)}^{i}(o^{\prime })-x_{+}^{i,j}\right]}^{T}}{M}) \end{array}$$

At this time,

(17) $$x_{+}^{i,j}∼f_{+}(x\left|x_{\left(k-1\right)}^{i,j},o^{\prime }\right.)$$

(18) $$x_{\left(k-1\right)}^{i,j}∼N(x;m_{\left(k-1\right)}^{i}(o^{\prime }),p_{\left(k-1\right)}^{i}(o^{\prime }))$$

(4) Update of GM-JMNS-CPHD filter

The update of the GM-JMNS-CPHD filter can be expressed as follows:

Given the distribution of prediction cardinality $p_{\left(k+1\left|k\right.\right)}(n)$ and $D_{\left(k+1\left|k\right.\right)}(x,o)$, the update function of PHD at time k can be expressed as follows:

(19) $$p_{\left(k\left|k\right.\right)}(n)=\frac{b_{k}^{0}\left[D_{\left(k+1\left|k\right.\right)},Z_{k}\right](n)p_{\left(k+1\left|k\right.\right)}(n)}{\langle b_{k}^{0}\left[D_{\left(k+1\left|k\right.\right)},Z_{k}\right],p_{\left(k+1\left|k\right.\right)}\rangle }$$

(20) $$D_{\left(k\left|k\right.\right)}(x,o)=(1-p_{\left(k\left|k\right.\right)}(x,o))\frac{\langle b_{k}^{1}\left[D_{\left(k+1\left|k\right.\right)},Z_{k}\right],p_{\left(k+1\left|k\right.\right)}\rangle }{\langle b_{k}^{0}\left[D_{\left(k+1\left|k\right.\right)},Z_{k}\right],p_{\left(k+1\left|k\right.\right)}\rangle }D_{\left(k+1\left|k\right.\right)}(x,o)+\sum_{z\in Z_{k}}\sum_{i=1}^{J_{0,+}}A_{k}^{i}(z,o)B_{z}(o)C_{z}^{i}(x,o)$$

(21) $$A_{k}^{i}(z,o)=\frac{1}{M^{\prime }}\sum_{j=1}^{M^{\prime }}\frac{g_{k}(z\left|x_{k}^{\left(i,j\right)},o\right.)N(x_{k}^{\left(i,j\right)};m_{+}^{i}(o),P_{+}^{i}(o))}{\pi_{k}^{i}(x_{k}^{\left(i,j\right)}\left|Z_{1:k-1},z,o\right.)}$$

(22) $$B_{z}(o)=p_{\left(k\left|k\right.\right)}(x,o)\frac{\langle 1,\kappa_{k}\rangle \langle b_{k}^{1}\left[D_{\left(k+1\left|k\right.\right)},Z_{k}\right],p_{\left(k+1\left|k\right.\right)}\rangle }{\kappa_{k}(z)\langle b_{k}^{0}\left[D_{\left(k+1\left|k\right.\right)},Z_{k}\right],p_{\left(k+1\left|k\right.\right)}\rangle }$$

(23) $$C_{z}^{i}(x,o)=D_{\left(k+1\left|k\right.\right)}^{i}(o)N(x;m_{k}^{i}(z,o),P_{k}^{i}(z,o)$$

(24) $$P_{K}^{i}(z,o)=\frac{\sum_{j=1}^{M^{\prime }}\frac{g_{k}(z\left|x_{k}^{\left(i,j\right)},o\right.)N(x_{k}^{\left(i,j\right)};m_{+}^{i}(o),P_{+}^{i}(o))}{\pi_{k}^{i}(x_{k}^{i,j}\left|Z_{1:k-1}\right.,z,o)}[m_{k}^{i}(z,o)-x_{k}^{\left(i,j\right)}]{\left[m_{k}^{i}(z,o)-x_{k}^{\left(i,j\right)}\right]}^{T}}{\sum_{j=1}^{M^{\prime }}\frac{g_{k}(z\left|x_{k}^{\left(i,j\right)},o\right.)N(x_{k}^{\left(i,j\right)};m_{+}^{i}(o),P_{+}^{i}(o))}{\pi_{k}^{i}(x_{k}^{\left(i,j\right)}\left|Z_{1:k-1}\right.,z,o)}}$$

At this time, $x_{k}^{i,j}∼\pi_{k}^{i}(\cdot \left|\left|Z_{1:k-1}\right.,z,o\right.)$.

### 2.3. Stochastic Outlier Selection

In the application field of data clustering and dimensionality reduction, data affinity is often used as a research tool. The essence of the SOS algorithm is to apply the concept of affinity to outlier selection to realize clustering [38,39,40].

**Figure 5:**
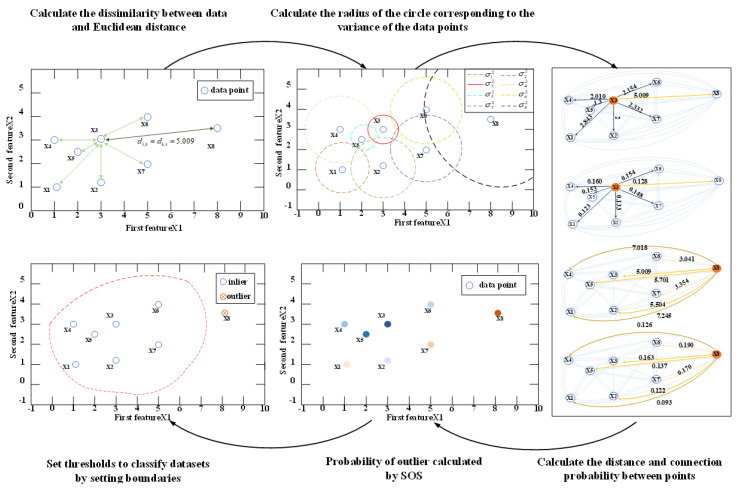
Schematic diagram of SOS algorithm.

The difference between the SOS algorithm and other methods is that this method is an unsupervised and unbounded outlier selection problem, which directly divides the data into “outlier” or “internal” values to realize the selection of outlier data [41,42,43]. The schematic diagram of the SOS algorithm is shown in Figure 5.

Illustrated in Figure 4, the process begins with inputting the matrix X containing the eigenvalues of data points. Initially, the dissimilarity matrix A is computed, representing the Euclidean distance between each feature point. Then, the affinity matrix B is independently calculated and combined with the probability matrix C to establish the joint distribution of various sequence row numbers. This amalgamation enables the estimation of the probability that a data point belongs to the outlier category. Subsequently, this probability serves as the outlier score within the SOS algorithm.

## 3. Application of SOS-GM-JMNS-CPHD Algorithm to Nonlinear Systems

Suppose the local strength of the sensor section is expressed as follows:

(25) $${\hat{D}}_{\left(x\right)}^{i}=\sum_{i=1}^{J^{i}}\alpha_{p}^{i}N(x;m_{p}^{i},P_{p}^{i})$$

Among them, i represents the number of sensors, and $\alpha_{p}^{i}\in (0,1)$ and N(x;m,P) represent Gaussian probability density functions (PDFs) with mean m and covariance p.

From the perspective of difference, different GCs may belong to different clusters $C_{g},g\in \left\{1,2,\cdots ,G\right\}$, which can be divided into two clusters, i and j. When in a cluster,

(26) $${\bar{D}}_{g}^{i,j}(x,o)={\hat{D}}_{g}^{i,j}(x,o)$$

The target number can be estimated according to the map standard. At this time, the cardinality distribution represented by g clusters is as follows:

(27) $${\bar{p}}_{g}^{i,j}(n)={\hat{p}}_{g}^{i,j}(n)=\prod_{p=1}^{M^{i,j}}\left(1-\alpha_{\left(g,p\right)}^{i,j}\right)\sigma_{M^{i,j},n}(\frac{\alpha_{\left(g,1\right)}^{i,j}}{1-\alpha_{\left(g,1\right)}^{i,j}},\cdots ,\frac{\alpha_{\left(g,M^{i,j}\right)}^{i,j}}{1-\alpha_{\left(g,M^{i,j}\right)}^{i,j}})$$

The three steps of splitting, fusion, and merging are discussed below.

### 3.1. Splitting

#### 3.1.1. Boundary Segmentation from Different Perspectives

**Figure 6:**
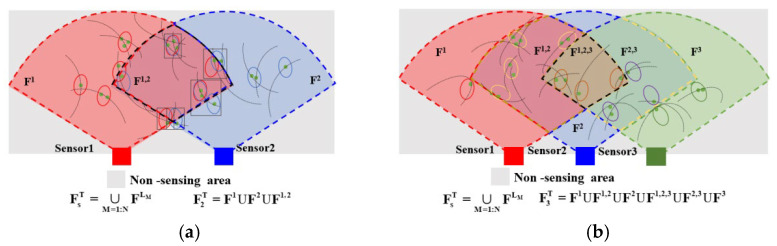
Different perspective scene division with different numbers of sensors. (**a**) Two sensor differential perspectives; (**b**) three sensor differential perspectives.

Figure 6 shows the view scene division in two cases of sensor difference views and three cases of sensor difference views.

As shown in Figure 5, the SOS-GM-JMNS-CPHD algorithm can divide the GCS near the boundary in the difference perspective, avoiding the problems of missing detection and the repeated calculation of GCs.

Among them,

(28) $$D_{g}^{i}(x,o)=1_{F^{LJ}}(x)D_{g}^{i}(,o)=\sum_{j=1:N}D_{g}^{j}(x,o)=\sum_{j=1:N}1_{X^{LJ}}(x)D_{g}^{i}(x,o)$$

At this time, the number of targets and the density of spatial targets segmented according to the perspective are as follows:

(29) $$M_{g}^{\left(i,j\right)}(x)=\sum_{o}\int_{x^{j}}D_{g}^{i}(x)dx$$

(30) $$s_{g}^{\left(i,j\right)}(x)=\frac{D_{g}^{i}(x)}{M_{g}^{\left(i,j\right)}(x)}$$

#### 3.1.2. SOS Clustering of GCs

$G_{p}^{i},G_{q}^{j}$ are GCs with different motion trajectories. According to the above SOS calculation process, it is determined that $\varphi SOS(G_{\left(p,q\right)}^{\left(i,j\right)})<\beta$ is divided into the same category. At this time, GCs are in the same cluster, and the node strength is as follows:

(31) $${\hat{D}}_{g}^{\left(i,j\right)}(x)=\sum_{g=1}^{G}{\hat{D}}_{g}^{\left(i,j\right)}(x)$$

$M_{g}^{\left(i,j\right)}$ indicates the number of GCs. Once $M_{g}^{\left(i,j\right)}>0$, it needs to be classified. The corresponding sub strengths are as follows:

(32) $${\hat{D}}_{g}^{\left(i,j\right)}(x)=\sum_{p=1}^{M_{g}^{\left(i,j\right)}}\alpha_{\left(g,p\right)}^{i}N(x;m_{\left(g,p\right)}^{i},P_{\left(g,p\right)}^{i})$$

Under the joint action of the classification method and the SOS clustering algorithm, boundary segmentation is performed to generate the number of targets ${M^{\prime }}_{g}^{\left(i,j\right)}(x)$ as follows:

(33) $${M^{\prime }}_{g}^{\left(i,j\right)}(x)=\sum_{o}\int_{x^{j}}{\hat{D}}_{g}^{i}(x)dx$$

The corresponding spatial target density and sub intensity can be expressed as follows:

(34) $$s_{g}^{\left(i,j\right)}(x)=\frac{{\hat{D}}_{g}^{i}(x)}{{M^{\prime }}_{g}^{\left(i,j\right)}(x)}$$

(35) $${\hat{D^{\prime }}}_{g}^{\left(i,j\right)}(x,o)=\sum_{p:(i,p)\in c_{g}}{\hat{D}}_{g}^{\left(i,j\right)}(x,o)=\sum_{p:(i,p)\in c_{g}}\alpha_{g,p}^{i}G(x,m_{\left(g,p\right)}^{i},P_{\left(g,p\right)}^{i})$$

Under the joint action of $F^{j}$ and C,$C^{\prime }$, the following is generated:

(36) $$\left({\left\{C_{g}\right\}}_{g=1}^{G}{)}^{\prime }\notin F_{s}^{T}(F_{s}^{T}=\underset{M=1:N}{\cup }F^{L_{M}}\right)$$

(37) $$C^{\prime }=({\left\{C_{g}\right\}}_{g=1}^{G}{)}^{\prime }$$

The above target RFS corresponding to $C^{\prime }$ is approximately Bernoulli, with a probability distribution of $G_{p}^{i}(x)$ and a probability of existence of $\alpha_{p}^{i}\in [0,1]$. The cardinality distribution formula for sensor i in $F^{j}$ is as follows:

(38) $${p^{\prime }}_{g}^{\left(i,j\right)}(n)={\hat{p}}_{g}^{\left(i,j\right)}(n)=\prod_{p=1}^{M^{i,j}}\left(1-\alpha_{\left(g,p\right)}^{\left(i,j\right)}\right)\sigma_{M^{\left(i,j\right)},n}(\frac{\alpha_{\left(g,1\right)}^{\left(i,j\right)}}{1-\alpha_{\left(g,1\right)}^{\left(i,j\right)}},\cdots ,\frac{\alpha_{\left(g,M^{i,j}\right)}^{\left(i,j\right)}}{1-\alpha_{\left(g,M^{i,j}\right)}^{\left(i,j\right)}})$$

At this point, $\sigma_{\left(M^{\left(i,j\right)},n\right)}(\cdot )$ is an N-th-order elementary symmetric function of $M^{\left(i,j\right)},n$.

According to the multi-Bernoulli MPD property, the cardinal number distribution of different regions is calculated as follows [44,45]:

(39) $$\sum_{n=0}^{\infty }np_{i}^{j}(n)=\sum_{m=1}^{M_{\left(i,j\right)}}\alpha_{p}^{i}={M^{\prime }}_{g}^{\left(i,j\right)}$$

(40) $$\sum_{n_{1}+n_{2}+\cdots n_{N}=n}\left[p_{i}^{1}(n_{1})\cdots p_{i}^{N}(n_{N})]=p_{i}(n\right)$$

### 3.2. Fusion

The GA fusion strategy is used to fuse N sensor networks.

(41) $$s_{g}^{\left(i.l+1\right)}(X)=\frac{\prod_{j\in Ni}{\left[s_{g}^{\left(i.l+1\right)}(X)\right]}^{\omega^{\left(i,j\right)}}}{\int \prod_{j\in N^{i}}{\left[s_{g}^{\left(i.l+1\right)}(X)\right]}^{\omega^{\left(i,j\right)}}dX}$$

(42) $$p_{g}^{\left(i,l+1\right)}(n)=\frac{\prod_{j\in N^{i}}{\left[p_{g}^{\left(i,l\right)}(n)\right]}^{\omega^{\left(i,j\right)}}{\left(\int \prod_{j\in N^{i}}{\left[s_{g}^{\left(j,l\right)}(X)\right]}^{\omega^{\left(i,j\right)}}dX\right)}^{n}}{\sum_{m=0}^{\infty }\prod_{j\in N^{i}}{\left[p_{g}^{\left(j,l\right)}(m)\right]}^{\omega^{\left(i,j\right)}}{\left(\int \prod_{j\in N^{i}}{\left[s_{g}^{\left(j,l\right)}(X)\right]}^{\omega^{\left(i,j\right)}}dX\right)}^{m}}$$

For each area, the cardinal distribution given by different sensors $p_{i}^{j}(n)$$D_{i}^{j}(x,o)$ is calculated, and the application of GA fusion strategy in CPHD is as follows:

(43) $$D_{GA}(x,o)=s_{GA}(x,o)\sum_{n=0}^{\infty }np_{GA}(n)$$

Among them,

(44) $$s_{GA}(x,o)=\frac{\sum_{m=0}^{\infty }\prod_{i\in N^{j}}{\left[s_{i}(x,o)\right]}^{\omega_{i}}}{\sum_{o}\int_{X}\prod_{i\in N^{j}}{\left[s_{i}(x,o)\right]}^{\omega_{i}}dx}$$

(45) $$p_{GA}(n)=\frac{\prod_{i\in N^{j}}{\left[p_{i}(n)\right]}^{\omega_{i}}{\left[\sum_{o}\int_{X}\prod_{i\in N^{j}}{\left[s_{i}(x,o)\right]}^{\omega_{i}}dx\right]}^{n}}{\sum_{m=0}^{\infty }\prod_{i\in N^{j}}{\left[p_{i}(m)\right]}^{\omega_{i}}{\left[\sum_{o}\int_{X}\prod_{i\in N^{j}}{\left[s_{i}(x,o)\right]}^{\omega_{i}}dx\right]}^{m}}$$

### 3.3. Merging

The fusion algorithm process of SOS-GM-JMNS-CPHD in multi-sensor and multi-perspective situations is shown in Algorithm 1.
**Algorithm 1:** SOS-GM-JMNS-CPHD fusion algorithm for multi-sensor and multi-perspective situations.  **Input:** ${\left\{G_{1,k}^{p}\right\}}_{p=1}^{M_{g}^{i}},{\left\{G_{2,k}^{p}\right\}}_{p=1}^{M_{g}^{j}}$, β, $C_{1}={\left\{\left(1,\;p\right)\right\}}_{p=1}^{M_{g}^{i}},C_{2}={\left\{\left(1,\;p\right)\right\}}_{p=1}^{M_{g}^{i}}$, $p_{i}^{j}(n)$, $F_{i}$Implementing filtering using GM-JMNS-CPHD
**for**
i∈N **do** **for**
j=1:N **do**  **for** $M_{g}^{i,j,k}=1:g$ **do**   $\mathrm{Locate}\;\mathrm{the}\;\mathrm{particles}\;\mathrm{situated}\;\mathrm{within}\;\mathrm{the}\;\mathrm{region}\;\mathrm{of}\;F^{j}$.   **for**
$(i^{\prime },p^{\prime })\in C$$\;\mathrm{and}\;(i^{\prime },p^{\prime })\neq (i,p)$ **do**
    $\mathrm{Calculate}\;C={\left\{C_{g}\right\}}_{g=1}^{G}$ by Algorithm 1
    $\mathrm{Calculate},\;({\left\{C_{g}\right\}}_{g=1}^{G}{)}^{\prime }\notin F_{s}^{T}(F_{s}^{T}=\underset{M=1:N}{\cup }F^{L_{M}})$$N_{k,g}=\left\{i:M_{g}^{i,j}>0\right\}\to N_{k,g}$$C^{\prime }=({\left\{C_{g}\right\}}_{g=1}^{G}{)}^{\prime }$    **end for**  **end for**  $\mathrm{Calculate}\;{\hat{D^{\prime }}}_{g}^{i,j}(x)$$\;\mathrm{by}\;(35),\;\mathrm{Calculate}\;{p^{\prime }}_{g}^{i,j}(n)$ by (38) **end for** **end for**  Calculate GA fusion  strategy by 43–45  $\mathrm{Calculate}\;\mathrm{cardinality}\;\mathrm{distribution}\;{\bar{p}}_{i}^{j}(n)$$\;\mathrm{and}\;\mathrm{fusion}\;\mathrm{target}\;\mathrm{state}\;\mathrm{density}\;{\bar{D}}_{g}^{i,j}(x)$  after merging by 46–47  
**Output:**
$\;{\bar{p}}_{i}^{j}(n)$
$,\;{\bar{D}}_{g}^{i,j}(x)$


To obtain the multi-target probability density distribution of GCs across various sensors and diverse perspectives, it is crucial to integrate the multi-target probability density distributions from the segmented regions discussed earlier. This involves consolidating all combined sub-IID cluster processes into a unified IID cluster process. This consolidation is accomplished by aggregating all sub-strength functions and convolving the corresponding cardinality distributions; the merged cardinality distributions ${\bar{p}}_{i}^{j}(n)$ and GM-JMNS-CPHD ${\bar{D}}_{g}^{i,j}(x)$ are as follows:

(46) $${\bar{D}}_{g}^{i,j}(x)=\sum_{g=1}^{G}{\bar{D^{\prime }}}_{g}^{i,j}(x)$$

(47) $${\bar{p}}_{i}^{j}(n)=({\bar{p}}_{1}*{\bar{p}}_{2}\cdots {\bar{p}}_{G})(n)=\sum_{n_{1}+n_{2}+\cdots +n_{G}=n}{\bar{p}}_{1}(n_{1}){\bar{p}}_{2}(n_{2})\cdots {\bar{p}}_{G}(n_{G})$$

## 4. Simulation Results

Using the CV model for simulation verification, the following is obtained:

(48) $$F_{k}=\left[\begin{array}{cc} I_{2} & \Delta I_{2}\\ O_{2} & I_{2} \end{array}\right]$$

(49) $$Q_{K}=\sigma_{v}^{2}\left[\begin{array}{cc} \frac{\Delta^{4}}{4}I_{2} & \frac{\Delta^{3}}{2}I_{2}\\ \frac{\Delta^{3}}{2}I_{2} & \Delta^{2}I_{2} \end{array}\right]$$

(50) $$H_{k}=[\begin{array}{cc} I_{2} & O_{2} \end{array}],\;R_{k}=\sigma_{\varepsilon }^{2}I_{2}$$

This section will employ a GM-JMNS-CPHD filter to evaluate the tracking performance of the SOS-GM-JMNS-CPHD algorithm proposed in the study and to validate its effectiveness. A nonlinear CT model will be used. The CT model assumes that the target undergoes a uniform turning motion in a two-dimensional plane, $CT(x)={\left(x_{k}\;y_{k}\;v_{x,k}\;v_{y,k}\right)}^{⊤}$ It can be described as the following matrix form:

The target status is as follows:

(51) X(k+1)=F(k)X(k)+Γ(k)w(k)

Among them,

(52) $$F=\left[\begin{array}{cccc} 1 & 0 & \frac{\sin\omega T}{\omega } & -(\frac{1-\cos\omega T}{\omega })\\ 0 & 1 & \frac{1-\cos\omega T}{\omega } & \frac{\sin\omega T}{\omega }\\ 0 & 0 & \cos\omega T & -\sin\omega T\\ 0 & 0 & \sin\omega T & \cos\omega T \end{array}\right],\Gamma (k)=\left[\begin{array}{cc} \begin{array}{c} 0.5T^{2}\\ 0\\ T\\ 0 \end{array} & \begin{array}{c} 0\\ 0.5T^{2}\\ 0\\ T \end{array} \end{array}\right],w(k)=\left[\begin{array}{c} w_{x}\\ w_{y} \end{array}\right]$$

### 4.1. Application of SOS-GM-CPHD in Nonlinear System Result Analysis

**Figure 7:**
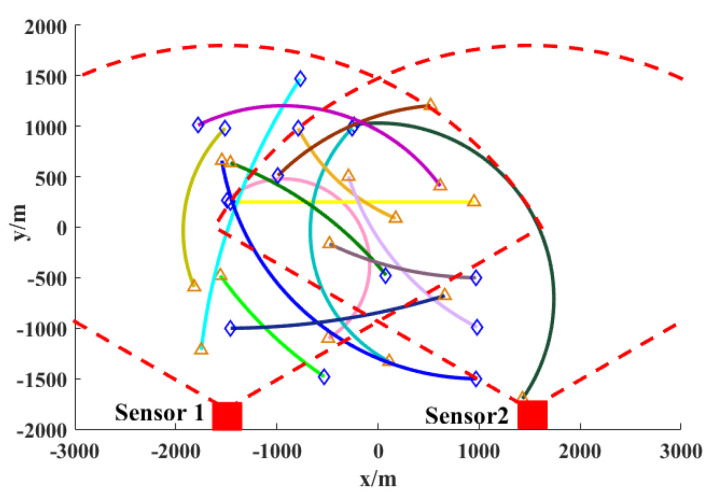
Application of SOS−GM−JMNS−CPHD in multi−target tracking of nonlinear systems.

To assess the algorithm’s performance, it was evaluated across various scenarios through simulation and comparative analysis. This study employed a GA fusion strategy to evaluate the tracking performance of the SOS-GM-JMNS-CPHD algorithm. Specifically, the algorithm was tested using 15 motion trajectories across two sensors to analyze the detection probabilities of different objects $p_{D}$, along with the Poisson average velocity value $\lambda_{c}$ of uniform clutter, across different scenarios. The goal was to evaluate their respective impacts on the algorithm’s performance. $\lambda_{c}=5,\;10,\;20,\;30$ and $p_{D}=0.50,\;0.65,\;0.85,\;0.95$ were set, and 120 Monte Carlo runs were performed. The application of SOS-GM-JMNS-CPHD in the multi-target tracking of linear systems is shown in Figure 7.

**Figure 8:**
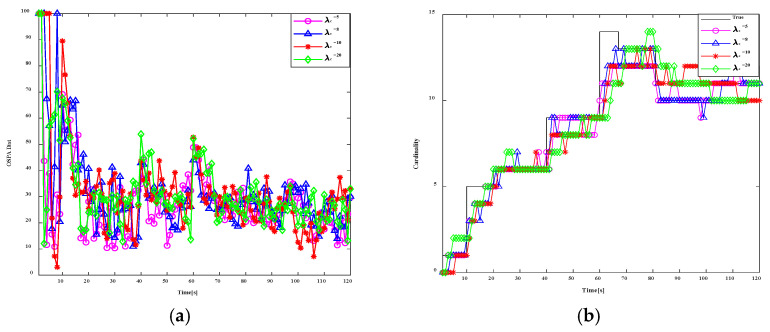
Comparison results of different SOS-GM-JMNS-CPHD $\lambda_{c}$.

**Figure 9:**
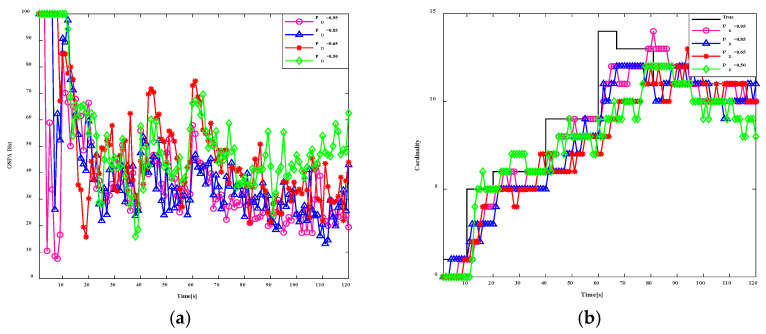
Comparison results of different SOS-GM-JMNS-CPHD $p_{D}$.

Similarly, in order to test the application of the algorithm to tracking moving targets in nonlinear systems, we assess the impact of the SOS-GM-JMNS-CPHD algorithm’s performance across 20 motion tracks involving 16 sensors at $\lambda_{c}=10$ and $p_{D}=0.50,\;0.65,\;0.85,\;0.95$. The results are shown in Figure 8 and Figure 9. It can be seen from the figures that the algorithm has a certain degree of robustness.

### 4.2. Comparative Analysis of SOS-GM-JMNS-CPHD and Other Algorithms

**Figure 10:**
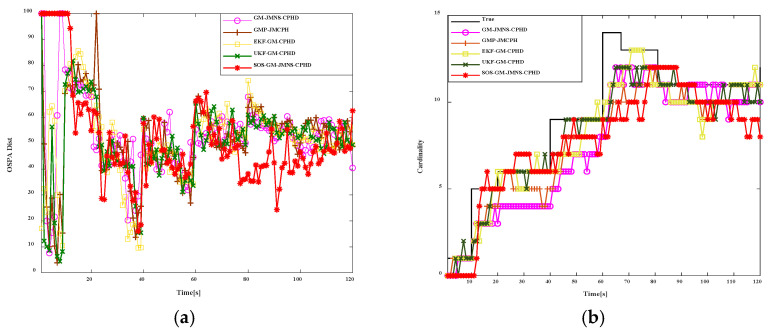
Comparison results of SOS-GM-JMNS-CPHD and different algorithms. (**a**) Different algorithms’ OSPA metrics over time. (**b**) Different algorithms’ cardinality estimates over time.

To evaluate the performance of this algorithm against others, this study implements and compares the filtering performance across several scenarios. The effectiveness of SOS-GM-JMNS-CPHD and GM-JMNS-CPHD and EKF-GM-CPHD and UKF-GM-CPHD is compared in references. We set up 15 motion trajectories for $p_{D}=0.95$, $\lambda_{c}=10$, and two sensors for research and analysis. Different algorithms’ OSPA (Optimal Sub-Pattern Assignment) metrics over time and different algorithms’ cardinality estimates over time are shown in Figure 10. The comparison results between SOS-GM-JMNS-CPHD and different algorithms show that the SOS-GM-JMNS-CPHD algorithm proposed in this study is significantly better than other algorithms due to its effectiveness. The research results are similar to GMP-JMCPHD and are more effective compared to other methods.

### 4.3. Algorithm Complexity

In general, the computation of the cardinal distribution is model-independent [46,47], as the calculation demand remains unaffected by the increase in the number of targets in the scenario. However, the computational complexity of the CPHD and SOS-GM-JMNS-CPHD algorithms is a factor to consider. The CPHD algorithm can be seen as evaluating an elementary pair production function m + 1 times, with a complexity of $O(m^{3})∼O{\left|Z\right|}^{3}n_{\max})$, where m is the number of evaluations and $n_{\max}$ is the maximum number of targets. When $n_{\max}>m$, CPHD can be considered as $\left(m^{3}+n_{\max}m^{2}-\frac{m^{2}}{2})∼O(n_{\max}m^{2}\right)$. As mentioned earlier, the computational complexity of JMNS-CPHD increases linearly with the number of patterns, from $O({\left|Z\right|}^{3}n_{\max})$ to $O({\left|Z\right|}^{3}n_{\max}O)$, where $n_{\max}$ is the maximum number of targets.

SOS-GM-JMNS-CPHD does not have any impact on the cardinality distribution of CPHD during use, but rather changes the maximum number of targets.

$n_{\max}\to {\bar{n}}_{\max}$ through clustering algorithms. Although clustering algorithms may increase algorithm difficulty, they also have an impact on the maximum number of targets. The complexity of the SOS-GM-JMNS-CPHD algorithm can be considered as $O({\left|Z\right|}^{3}n_{\max}O+O)∼O({\left|Z\right|}^{3}n_{\max}O)$, and the complexity of the algorithm will not occur linearly with the increase in the number of targets. Compared with CPHD, GM-JMNS-CPHD, and SOS-GM-JMNS-CPHD, the complexity of the three algorithms is $O_{CPHD}<O_{GM-JMNS-CPHD}=O_{SOS-GM-JMNS-CPHD}$.

## 5. Conclusions

This paper presents a novel approach for random anomaly selection in distributed multi-sensor fusion, tailored for nonlinear systems with varying view angles. The method combines the SOS clustering algorithm with the GM-JMNS-CPHD filtering technique. By delineating boundaries based on different perspectives, the approach divides them into disjoint segmentation regions. SOS is then employed to identify outliers, ensuring that the split cardinal distribution accurately reflects the target distribution in each corresponding region, while preserving the original cardinal distribution. The implementation of a distributed multi-sensor fusion method for nonlinear systems with diverse perspectives is realized. This method addresses issues such as repeated detection and local density loss due to fields of view (FOVs) in distributed fusion setups, which often lead to the overestimation or underestimation of target numbers. Consequently, it enhances the accuracy of local density acquisition within FOVs. The robustness and effectiveness of this approach are confirmed through simulation results, showcasing its superiority over alternative methods. Additionally, an analysis of algorithm complexity reveals that it does not increase linearly with the number of targets.

This paper proposes a distributed fusion random outlier selection method for sensor networks with different fields of view. The research and practice in this field is only a guide. There are many directions worthy of in-depth research in the future, such as technological breakthroughs in complex scenes; in the actual distributed fusion technology of sensor networks, there are many complex scenes, such as the sensor node acquisition clock not being synchronized, the sensor position being unknown, or the angle of view being unknown, and there are time and angle differences, as well as heterogeneous sensor configuration, and the sensor network itself is subject to strong interference and instability, and so on. In the follow-up study, we can break through the above practical and challenging technical problems one by one.

## Data Availability

Data are contained within the article.
